# Physiological adaptation during heart rate variability biofeedback in young adults: A survival analysis in high‐stress academic environments

**DOI:** 10.14814/phy2.70949

**Published:** 2026-06-04

**Authors:** Gabriela Panayotova, Margarita Velikova

**Affiliations:** ^1^ Division of Physiology, Department of Physiology and Pathophysiology Medical University of Varna Varna Bulgaria

**Keywords:** anxiety, biofeedback, depression, emWave, HRV, stress

## Abstract

Medical education is a high‐demand environment where stress may disrupt autonomic and HPA‐axis regulation. We examined symptom trajectories and adaptation during heart rate variability biofeedback (HRV‐BF) in an academic setting. Forty‐seven students were followed for ~3 months. The intervention group completed HRV‐BF twice weekly (~30 min/session). Psychological outcomes (PSS, SAS, BAI, BDI) were assessed at baseline and follow‐up, with clinically meaningful improvement defined as a ≥ 20% reduction. HRV/coherence indices were analyzed with mixed‐effects models, and cortisol and secretory IgA were measured around Maastricht Acute Stress Test before and after training. Within the HRV‐BF group, perceived stress, anxiety, and depression declined by May (all *p* < 0.001; *d*≈1.28–1.86), although between‐group differences converged. Meaningful improvement was more frequent in HRV‐BF group across scales. Acute sessions increased coherence and inter‐beat interval and reduced heart rate (*p* ≤ 0.015), while RMSSD and inter‐beat interval gains plateaued after ~14 sessions. Cortisol responses to stress differed after training, whereas sIgA showed no between‐group effects. In this nonrandomized study, HRV‐BF was associated with clinically meaningful improvement by follow‐up and autonomic adaptation; however, because the group was not matched on baseline distress severity and no sham comparator was used, reductions in anxiety and depression cannot be attributed definitively to the intervention.

## 1 INTRODUCTION

Medical education represents a sustained, high‐demand environment in which academic pressure, sleep disruption, and performance expectations converge into persistent psychophysiological stress (Benítez‐Agudelo et al., 2025; Santiago et al., 2024). This burden may be amplified in international medical students, who often experience additional adaptation demands related to acculturation, disrupted social support, and altered health behaviors (Jumaa et al., 2025). In this context, interventions that are low‐cost, scalable, and compatible with students' schedules are attractive, yet their physiological impact and the temporal dynamics of response remain incompletely characterized (Bennett‐Weston et al., 2024).

From an applied physiology perspective, stress‐related symptom trajectories are not purely psychological; they reflect coordinated responses across stress‐regulatory systems, including autonomic regulation and hypothalamic–pituitary–adrenal (HPA) axis activity (Chu et al., 2026). Heart rate variability (HRV), particularly vagally mediated indices, provides a noninvasive marker of cardiac autonomic regulation, whereas salivary cortisol is widely used to index HPA‐axis activation (Becker et al., 2020; McCraty & Shaffer, 2015; Sloan et al., 2017). Secretory immunoglobulin A (sIgA) complements these measures by reflecting mucosal immune function that can vary with stress exposure and regulatory capacity (Li et al., 2020; Mantis et al., 2011). Physiological stress responsivity is commonly characterized by anchoring repeated salivary cortisol and sIgA measurements to a validated acute stress challenge (Hellhammer et al., 2009; Mendes et al., 2022). Sampling across pre‐stress, immediate post‐stress, and recovery intervals allows investigators to distinguish basal tone from stress‐evoked reactivity and recovery kinetics (Dogan et al., 2022).

HRV biofeedback (HRV‐BF) and cardiac coherence training are practical, physiology‐oriented approaches intended to strengthen self‐regulation by pairing paced breathing and attentional strategies with real‐time feedback on cardiac rhythm dynamics (Blum et al., 2019; Lalanza et al., 2023; Lin et al., 2020). Training is typically delivered through platforms that derive inter‐beat intervals from photoplethysmography or electrocardiography (ECG) signals and compute HRV and coherence‐related indices, enabling repeated quantification of autonomic regulation over time (Shaffer & Meehan, 2020; Tsai et al., 2025). During training bouts, participants often exhibit shifts consistent with increased parasympathetic modulation and greater cardiorespiratory coupling, such as reduced heart rate, prolonged RR intervals, and higher coherence (Addleman et al., 2024; Andrade et al., 2020; Michael et al., 2017). Repeated induction of these regulated states across sessions is commonly conceptualized as a skill‐acquisition process in autonomic regulation with potential relevance for stress responsivity and affective symptom trajectories (Pasquini et al., 2023).

Despite growing use of HRV‐BF, most studies emphasize average pre–post change, which can obscure heterogeneity in responsiveness and, critically, the tempo of improvement (Castro Ribeiro et al., 2023; Firth et al., 2022; Yoo et al., 2022). The speed with which individuals reach a clinically meaningful threshold can be as practically informative as the final magnitude of change because it informs personalization, adherence strategies, and resource allocation (Shaffer & Ginsberg, 2017; Tiwari et al., 2021). Time‐to‐event analysis provides a rigorous framework for quantifying response tempo in longitudinal intervention studies and accommodates censoring when improvement is not observed in all participants during follow‐up (Korn et al., 1997; Ranganathan et al., 2025). Improvement can be operationalized using predefined criteria on validated measures (e.g., ≥20% symptom reduction from baseline), after which Kaplan–Meier methods summarize event‐time distributions and Cox proportional hazards models evaluate between‐group differences and associations with baseline predictors (Moore, n.d.; Pocock et al., 2002; Schober & Vetter, 2018).

The present article extends this framework from identifying whether improvement occurs to identifying physiological correlates of earlier improvement. Specifically, we examine time‐to‐improvement during HRV‐BF training in international medical students and test whether baseline autonomic regulation and acute stress physiology—indexed by HRV measures across training and salivary cortisol/sIgA dynamics surrounding the Maastricht Acute Stress Test (MAST)—are associated with earlier attainment of clinically meaningful symptom reduction. We hypothesize that a more favorable baseline autonomic profile, reflected by HRV‐related measures such as lower heart rate, longer inter‐beat interval, and/or higher RMSSD and coherence and a more adaptive stress‐response pattern, reflected in endocrine reactivity and recovery (with potential immune contributions), will be associated with shorter time‐to‐improvement during HRV‐BF training.

## 2 METHODS

### 2.1 Study design and setting

A controlled, parallel‐group intervention study was conducted with two arms: HRV biofeedback training and a no‐intervention observational control. Participants were international medical students enrolled in the Medicine program at Medical University “Prof. Dr. Paraskev Stoyanov” – Varna, Bulgaria. Group allocation was nonrandom: students meeting prespecified screening criteria for elevated stress and/or affective symptoms on baseline questionnaires were allocated to the HRV biofeedback arm, whereas a concurrently recruited comparison group was followed without intervention to characterize stress‐related outcomes in the absence of external support. The observation period was approximately 3 months per participant. The training group attended supervised laboratory sessions twice weekly. The control group did not receive a placebo or sham intervention; it served as a no‐intervention observational comparison arm followed over the same study period. The intervention and follow‐up took place during the active summer semester. According to the official academic calendar of Medical University – Varna for the relevant study year, the summer semester ran from 12 February 2024 to 23 May 2024, whereas the regular summer examination session began on 30 May 2024. Thus, the final assessments were obtained before the onset of the regular examination period and before summer vacation. Because participants were recruited from different academic years, differences in curriculum‐related workload across study years could not be fully standardized and remain a potential source of residual confounding. During the follow‐up period, participants were asked about major changes in mental health care. No participant reported initiation of anxiolytic or antidepressant medication, and no participant reported seeking formal mental health counseling during the study period.

### 2.2 Ethics, confidentiality, and informed consent

All participants were volunteers and received no financial or other compensation. Confidentiality procedures were implemented, including secure storage of study documentation. Written informed consent was obtained from all participants, with explicit permission to withdraw at any time without adverse consequences. Ethical approval for the study was granted by the Ethics Committee for Scientific Research at Medical University “Prof. Dr. Paraskev Stoyanov” – Varna (Protocol No. 137/02.11.2023). The study was conducted in accordance with the principles of the Declaration of Helsinki (2013).

### 2.3 Participants

A total of 47 international medical students participated: 24 in the HRV biofeedback training group and 23 in the control group (Table 1).

**TABLE 1:** Sociodemographic characteristics of the participants (*N* = 47).

Variable	Overall (*N* = 47)	HRV‐BF group (*n* = 24)	Control group (*n* = 23)
Sex, *n* (%)			
Male	7 (14.9)	3 (12.5)	4 (17.4)
Female	40 (85.1)	21 (87.5)	19 (82.6)
Age, years	23.09 ± 3.02	21.88 ± 1.68	24.35 ± 3.59
Weight, kg	69.79 ± 15.57	68.13 ± 15.43	71.52 ± 15.86
Height, cm	167.41 ± 9.12	166.50 ± 7.90	168.35 ± 10.34
Academic year, *n* (%)			
1	4 (8.5)	4 (16.7)	0 (0.0)
2	11 (23.4)	8 (33.3)	3 (13.0)
3	20 (42.6)	5 (20.8)	15 (65.2)
4	5 (10.6)	0 (0.0)	5 (21.7)
5	6 (12.8)	6 (25.0)	0 (0.0)
6	1 (2.1)	1 (4.2)	0 (0.0)

*Inclusion criteria* comprised:

- enrollment in the international “Medicine” program at the Medical University of Varna, Bulgaria;
- voluntary interest after initial screening for stress/anxiety/depression;
- moderate‐to‐high stress, anxiety, and/or depressive symptoms based on standardized psychometric questionnaires (PSS, SAS, BAI, BDI).

*Exclusion criteria* included:

- voluntary discontinuation of the training program;
- acute illness at program start;
- chronic conditions that prevented regular attendance and completion of the training protocol

For baseline comparability analyses, psychiatric conditions and chronic conditions were coded as self‐reported dichotomous variables (present/absent) and analyzed as nominal factors together with sex, smoking, and alcohol use. Baseline comparability on key nominal demographic/behavioral variables was evaluated using *χ* ^2^ testing, supporting general group homogeneity across factors such as sex, smoking, alcohol use, and chronic/psychiatric conditions (Table 2).

**TABLE 2:** Baseline comparability between groups using *χ* ^2^ testing.

Baseline factor	Statistic	*p*
Sex	0.22	0.638
Smoking	2.05	0.359
Alcohol use	1.12	0.571
Psychiatric conditions	1.47	0.481
Chronic conditions	2.70	0.101

*Note*: Psychiatric conditions and chronic conditions were coded as self‐reported present/absent variables at baseline; *χ* ^2^ statistics compare category frequencies between groups.

### 2.4 HRV biofeedback training

The training group received HRV biofeedback aimed at improving autonomic self‐regulation and heart rhythm coherence using the emWave® Pro Plus system (HeartMath Institute). Supervised laboratory sessions were conducted twice weekly and lasted approximately 30 min each. The system used an optical photoplethysmography (PPG) finger sensor to acquire pulse waveforms and derive beat‐to‐beat intervals for real‐time HRV feedback. The software environment also includes an HRV assessment module that provides spectral representations of the mean inter‐beat interval (IBI) signal and software‐derived frequency‐domain indices, from which LF and HF values used in exploratory analyses were obtained.

During training, the software displayed heart rate, coherence level, HRV tachograms, and spectral information. The intervention incorporated structured HeartMath coherence‐building exercises delivered with real‐time biofeedback. Heart‐focused breathing referred to directing attention to the heart/chest area while breathing slightly more slowly and deeply than usual in a comfortable rhythm; this constituted the basic attentional‐breathing component of training. No fixed inspiratory/expiratory ratio or respiratory frequency was imposed; instead, participants were guided to breathe in a slow, comfortable, individually self‐paced manner while maintaining attention on the heart/chest area and observing the real‐time feedback. Quick Coherence referred to a brief guided self‐regulation exercise that combined heart‐focused breathing with the intentional generation of a positive or renewing emotional state, such as appreciation, care, calmness, or ease, while participants observed their real‐time feedback. Heart Lock‐In referred to a longer, sustained version of the same practice, in which participants attempted to maintain heart‐focused attention together with a positive emotional state over several minutes in order to stabilize and reinforce coherent heart rhythm patterns.

The software also provided real‐time visual and interactive feedback modules. The Coherence Coach was the guided training interface that taught and paced the Quick Coherence procedure using narration, music, animation, and an adjustable breathing pacer. The balloon animation and garden were software visualizers that translated moment‐to‐moment coherence performance into intuitive visual displays, thereby supporting engagement and skill acquisition during the sessions. Participants were also encouraged to practice independently at home (e.g., after waking, before sleep, during exam preparation, and before anticipated stressors), but only supervised laboratory sessions were included in the longitudinal analyses.

### 2.5 HRV signal acquisition and processing

HRV data were acquired with the emWave® Pro Plus system using an optical photoplethysmography finger sensor. According to the device documentation, pulse‐wave sampling is performed at 370 Hz, with automatic pulse‐wave detection and calibration. During each supervised visit, HRV assessments were recorded for 5 min immediately before and 5 min immediately after the training session, using identical sensor placement and acquisition settings across visits. Interbeat interval data were visually reviewed within the software environment, and artifact editing was performed as needed before HRV calculation.

### 2.6 HRV measures during training

During each supervised training visit, HRV recordings were obtained 5 min before and 5 min after the session using the same sensor placement and acquisition settings. These paired pre‐session/post‐session measurements were used to quantify acute within‐session physiological change. The HRV variables included mean heart rate (MHR), mean inter‐beat interval (IBI), heart rate range, standard deviation of normal‐to‐normal intervals (SDNN), root mean square of successive differences (RMSSD), and normalized coherence. In the present study, normalized coherence refers to the HeartMath‐derived coherence index obtained from spectral analysis of the IBI/HRV signal. This index reflects the extent to which heart rhythm power is concentrated around a dominant peak within the coherence band of the HRV spectrum, relative to total spectral power. Because no separate respiratory channel was recorded, coherence was not calculated as formal cross‐spectral coherence between respiration and heart rate signals. In addition to the time‐domain HRV variables and normalized coherence, exploratory conventional frequency‐domain indices were extracted from the software‐derived spectral output of the IBI signal, including low‐frequency (LF) and high‐frequency (HF) components. These indices were not used as primary longitudinal endpoints across training sessions but were retained for secondary exploratory association analyses involving biomarker and psychometric change variables (Table 10). Because respiration was not recorded separately and breathing was intentionally slowed during training, LF and HF findings were interpreted cautiously and were not treated as direct measures of respiration‐independent autonomic balance or formal cardiorespiratory coherence.

### 2.7 Psychological assessments

**FIGURE 1:**
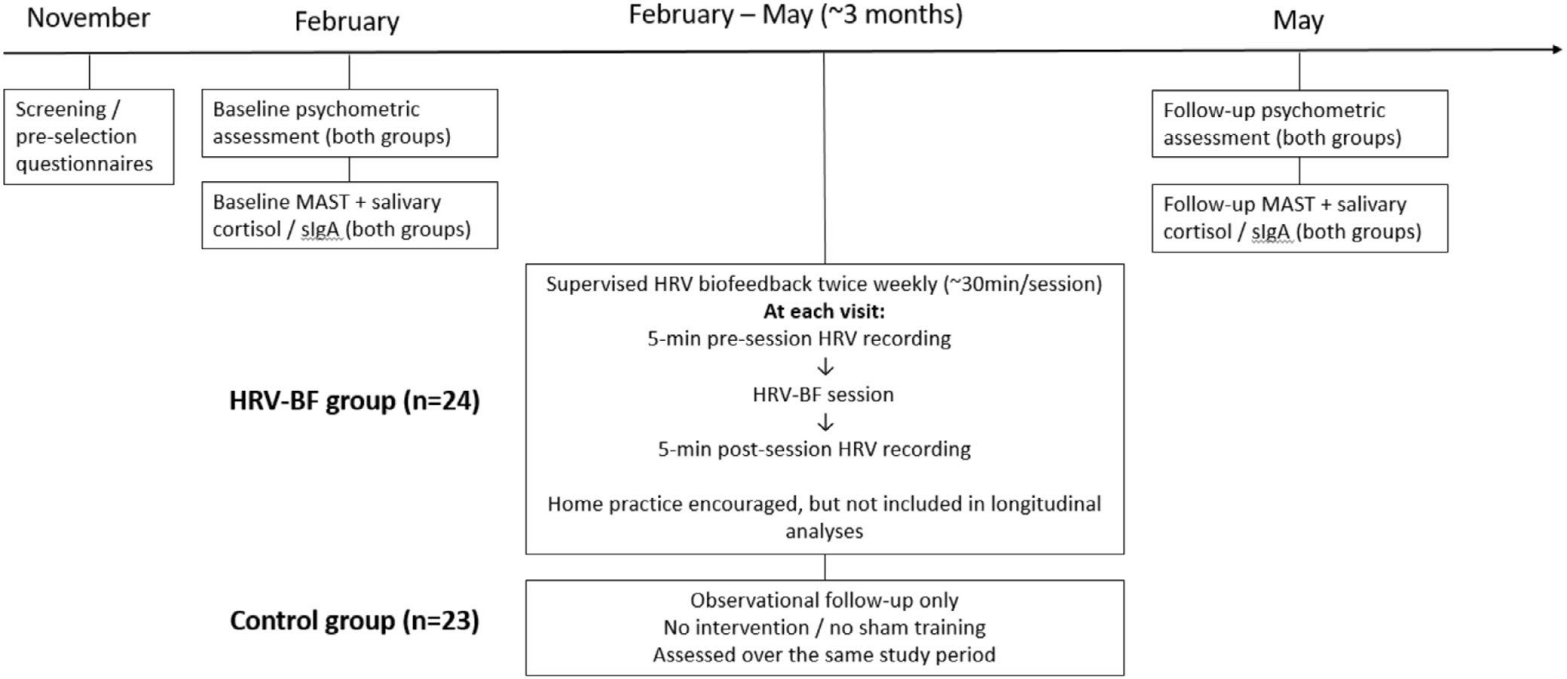
Study protocol and timing of assessments in the HRV biofeedback and control groups.

Psychological status was assessed using four standardized questionnaires (Perceived Stress Scale (PSS), Zung Self‐Rating Anxiety Scale (SAS), Beck Anxiety Inventory (BAI), and Beck Depression Inventory (BDI)) conducted in three defined time windows (Figure 1):

- November: screening/pre‐selection;
- February: baseline immediately before intervention start;
- May: post‐intervention at the end of the training program

The PSS is a widely used psychological instrument for measuring the perception of stress. It assesses the degree to which respondents find their lives unpredictable, uncontrollable, and overloading. The overall scores can be evaluated as follows: 0–13 – low stress; 14–26 – moderate stress; 27–40 – high perceived stress (Cohen et al., 1983).

The BDI is a 21‐item self‐report inventory designed to measure characteristic attitudes and symptoms of depression. The total score is classified as follows: 17–20 indicates borderline clinical depression, 21–30 moderate depression, 31–40 severe depression, and scores above 40 suggest extreme depression (Beck et al., 1961).

The SAS is a 20‐item self‐report scale that evaluates the severity of anxiety symptoms developed in the past several days. The raw score is converted into an index score, with values above 45 suggesting clinically significant anxiety (Zung, 1971).

The BAI consists of 21 multiple‐choice self‐report inventory questions to measure characteristic attitudes and symptoms of anxiety. Scores between 0 and 21 indicate low anxiety, 22–35 moderate anxiety, and scores above 36 suggest potentially severe anxiety (Beck et al., 1988).

For the purposes of the intervention analysis, psychometric outcomes were evaluated longitudinally across the approximately 3‐month study period using the baseline (February) and follow‐up (May) assessments.

### 2.8 Acute stress induction and salivary biomarkers

To examine psychophysiological stress reactivity, the Maastricht Acute Stress Test (MAST) was used as a standardized acute laboratory stressor. The protocol lasted ~10 min and alternated cold pressor exposure (0°C–4°C) with mental arithmetic evaluation (counting loudly backward from 2047 by 17), delivered in 10 sequential segments.

Salivary cortisol and secretory IgA (sIgA) were assessed on two laboratory testing days: once before the start of the training program and once after its completion. On each testing day, unstimulated saliva was collected at four predefined time points around the Maastricht Acute Stress Test (MAST):

- immediately after waking (rest);
- immediately before MAST;
- 10 min after MAST;
- 30 min after MAST.

Thus, each participant contributed repeated salivary measurements allowing characterization of basal level, acute stress reactivity, and early post‐stressor recovery.

Participants provided unstimulated saliva via passive drool into sterile containers, following standard pre‐collection restrictions (no food or drink except water, smoking, or tooth brushing within 30 min before sampling; seated calmly for ≥5 min). The target volume was ≥1 mL per sample. Samples were stored at −20°C until enzyme‐linked immunosorbent assay (ELISA) analysis. Salivary cortisol was assayed using a Cortisol Saliva ELISA Free kit (LDN Labor Diagnostika Nord GmbH & Co. KG, Nordhorn, Germany; Cat. No. SA E‐6000/SA E‐6000R), whereas secretory IgA was assayed using an IgA Saliva ELISA kit from the same manufacturer (Cat. No. SA E‐6800/SA E‐6800R).

### 2.9 Definition of “time‐to‐improvement physiology”

#### 2.9.1 Primary time‐to‐event endpoint (physiological)

A physiological improvement event was defined as the first point during the training program at which a participant showed a reliable, repeatable shift toward an autonomic pattern consistent with increased parasympathetic modulation, reflected by reduced heart rate, increased inter‐beat interval, and improved HRV/coherence measures during or after training sessions, relative to their own starting level. To establish each participant's starting level (“early‐training reference”), the analysis used their HRV‐related values from the initial phase of training as the within‐person comparator. Improvement was then evaluated using session‐level markers that have straightforward physiological interpretation—lower mean heart rate, higher mean inter‐beat interval, and/or higher normalized coherence. To ensure that the event reflected a stable change rather than day‐to‐day fluctuation, improvement was considered achieved only when the criterion was met on consecutive sessions. Participants who did not meet the criterion within the observation window were treated as censored in time‐to‐event analyses.

#### 2.9.2 Secondary clinical endpoint (follow‐up responder status)

A secondary clinical endpoint was defined as attainment of a clinically meaningful improvement threshold (≥20% reduction from baseline) on each psychometric scale (PSS, SAS, BAI, BDI). Because symptom scales were assessed at baseline and at the end of the intervention period, this endpoint was evaluated as improvement status by follow‐up (improved vs. not improved), rather than as a continuously observed event time.

In this study, autonomic adaptation was interpreted on the basis of measured cardiac autonomic markers, including heart rate, inter‐beat interval, RMSSD, and device‐derived coherence. Similarly, HPA‐axis response was interpreted from the salivary cortisol profile across repeated measurements obtained before and after the acute stress challenge.

### 2.10 Statistical analysis

Analyses were conducted, using:

- *χ* ^2^ tests for nominal baseline variables;
- normality checks (Shapiro–Wilk) and appropriate parametric/nonparametric group comparisons for continuous variables when applicable;
- mixed‐effects modeling of session‐level HRV indices using restricted maximum likelihood (REML) estimation with participant‐level random effects, consistent with the repeated‐measures structure and documented intraclass correlation.

Session‐level HRV outcomes were analyzed using linear mixed‐effects models to account for the repeated‐measures structure of the supervised training sessions, with participant included as a random effect. Acute within‐session responses were evaluated from the paired pre‐session/post‐session measurements. For longitudinal adaptation, both linear and nonlinear session trends were examined where appropriate. Salivary biomarker features used in time‐to‐event models were derived from the repeated sampling curves obtained on the pre‐training and post‐training MAST testing days, including basal/rest values, pre‐stressor levels, and post‐stressor change/recovery characteristics. Exploratory frequency‐domain indices (ΔLF and ΔHF) were not included in the primary mixed‐effects longitudinal models and were examined only in secondary within‐group correlation analyses.

#### 2.10.1 Survival modeling

Time‐to‐event outcomes were summarized using Kaplan–Meier curves and modeled using Cox proportional hazards regression for the physiological improvement endpoint derived from repeated session‐level HRV data. In contrast, clinically meaningful improvement on the psychometric scales was analyzed as responder status at follow‐up (May), because these outcomes were assessed only at baseline and end of follow‐up. Covariates considered included age and sex and a set of behavioral/health factors (e.g., smoking, alcohol use, financial status, parental support, chronic disease status, psychiatric history, medication/supplement use, sleep and study hours), acknowledging that small subgroup sizes can lead to unstable hazard ratio estimates. Menstrual cycle phase, hormonal contraception use, and other sex hormone–related variables were not recorded and therefore were not available for adjustment in the present analyses.

Physiological predictors for the Cox models included baseline HRV indices, acute session response metrics (Post–Pre contrasts), and salivary biomarker features derived from the MAST day curves (e.g., pre‐test level and post‐stressor changes), leveraging the repeated biomarker time points.

#### 2.10.2 Multiplicity and model diagnostics

For per‐session contrasts and multiple comparisons, multiplicity control was applied where relevant (e.g., Holm adjustment noted as impacting the number of “significant sessions” retained). Mixed‐model diagnostics included residual normality checks and residual–fitted pattern inspection; mild heteroskedasticity was noted, but model validity was supported overall.

## 3 RESULTS

### 3.1 Psychometric outcomes across the 3‐month intervention period (February → may)

**FIGURE 2:**
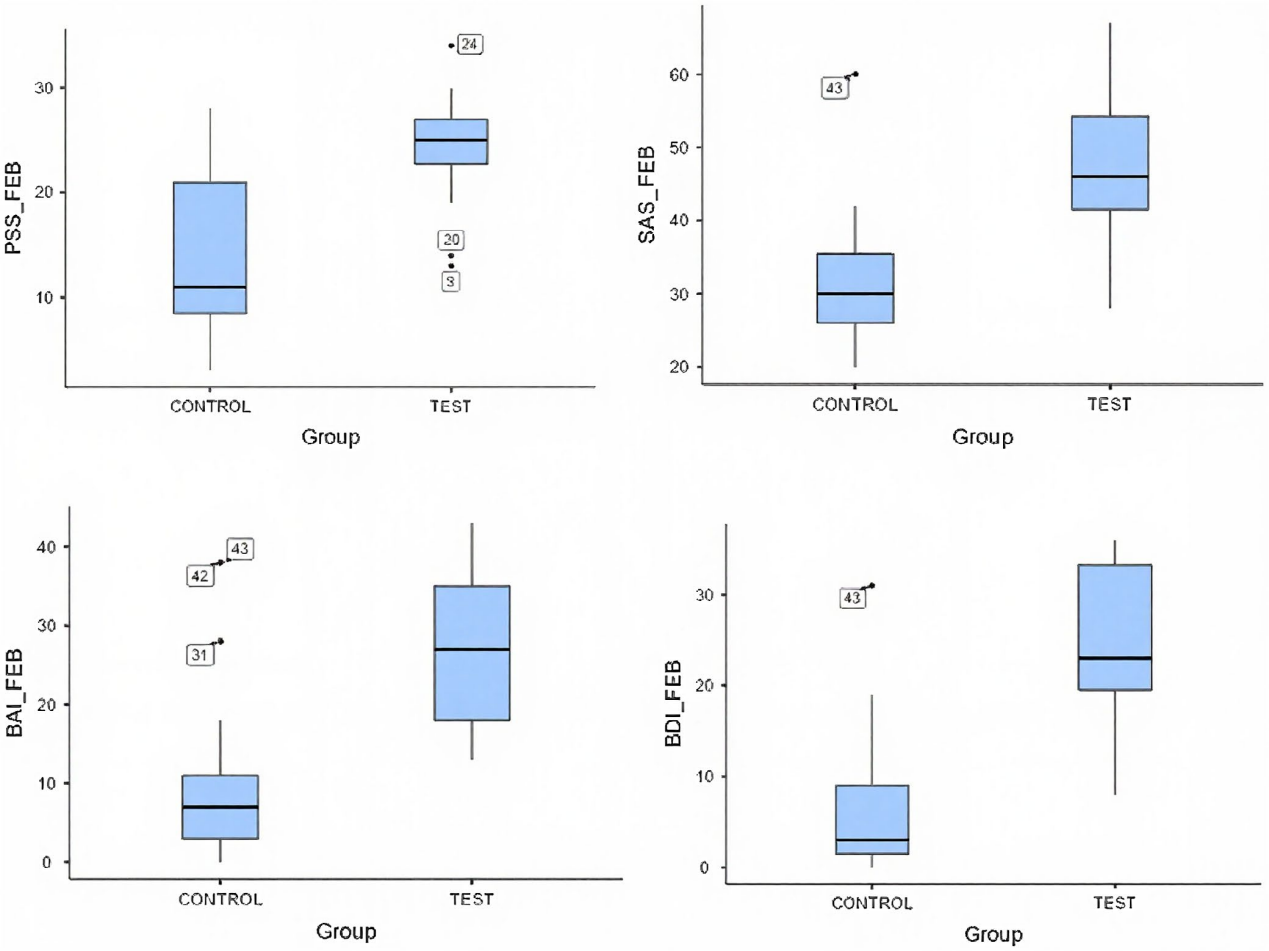
Baseline (February) psychometric scale scores by group (PSS, SAS, BAI, and BDI). Boxplots show the median (horizontal line), interquartile range (box), and minimum/maximum non‐outlier values (whiskers); individual outliers are plotted separately.

**FIGURE 3:**
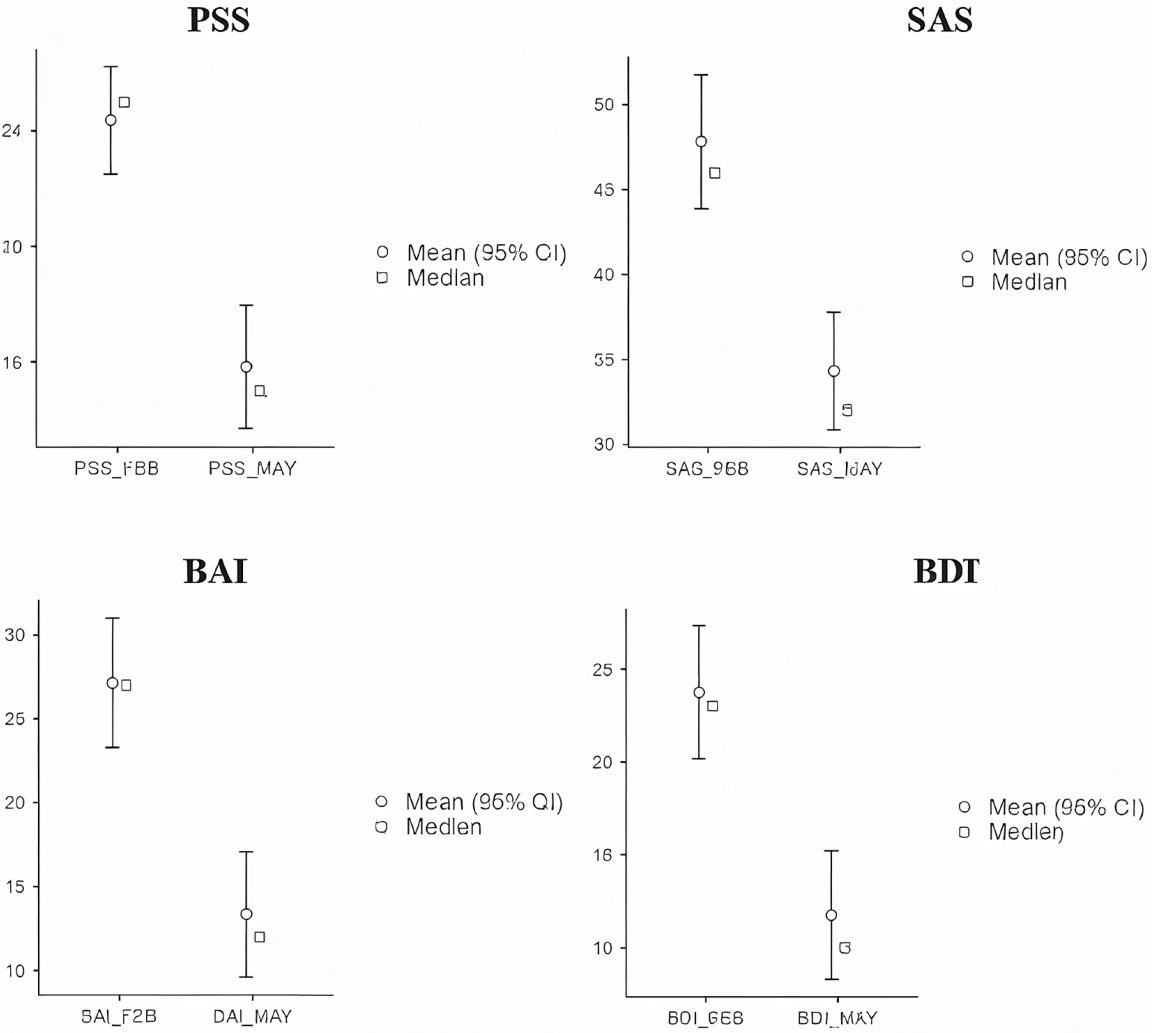
Mean (95% CI) and median psychometric scale scores at baseline and post‐training in the HRV biofeedback group (PSS, SAS, BAI, and BDI).

At baseline (February), the training group exhibited substantially higher distress across all psychometric endpoints relative to controls, indicating a markedly higher initial symptom burden (Figure 1 and Table 3). Formal between‐group comparisons confirmed statistically significant baseline differences for all four psychometric measures. Across the approximately 3‐month intervention period, these between‐group differences became markedly attenuated, and by May most scales no longer showed statistically significant separation between the HRV biofeedback and control groups (Table 3). Within the HRV biofeedback group, pre‐to‐post comparisons demonstrated robust improvements across all psychometric measures, with large effect sizes and consistently strong statistical support (*p* < 0.001 across endpoints, Table 4). Mean reductions were observed for perceived stress, anxiety, and depression, indicating clinically meaningful change over the intervention period (Figures 2 and 3).

**TABLE 3:** Psychometric scale scores by group at baseline (February) and post‐intervention (May), with between‐group comparisons.

Measure	Time point	Control mean ± SD	Training mean ± SD	*t*(df)	*p* (*t‐*test)	Cohen's *d*	*p* (*U* test)	Rank‐biserial corr.
PSS	February	13.70 ± 7.34	24.38 ± 4.62	−5.993 (45)	<0.001^*^	−1.75	<0.001^*^	0.7844
	May	15.43 ± 8.56	15.83 ± 5.33	−0.192 (45)	0.848	−0.056	0.717	0.0634
SAS	February	31.48 ± 8.56	47.83 ± 9.81	−6.079 (45)	<0.001^*^	−1.77	<0.001^*^	0.8207
	May	32.48 ± 9.88	34.33 ± 8.62	−0.687 (45)	0.496	−0.200	0.322	0.1703
BAI	February	10.00 ± 10.86	27.17 ± 9.65	−5.734 (45)	<0.001^*^	−1.67	<0.001^*^	0.7880
	May	10.91 ± 11.11	13.38 ± 9.31	−0.825 (45)	0.414	−0.240	0.114	0.2699
BDI	February	6.04 ± 7.22	23.75 ± 8.92	−7.462 (45)	<0.001^*^	−2.18	<0.001^*^	0.8768
	May	8.09 ± 9.16	11.75 ± 8.67	−1.408 (45)	0.166	−0.410	0.051	0.3333

^*^ *p* < 0.05.

**TABLE 4:** Within‐intervention group changes in psychometric scale scores from February to May: Paired *t*‐tests, Wilcoxon signed‐rank tests, and effect sizes.

Measure	Time point	Mean	SD	*t*(df)	*p*	Wilcoxon *W*	Cohen's *d*	Rank‐biserial corr.
PSS	February	24.4	4.62	6.62 (23)	<0.001	275	1.35	0.989
	May	15.8	5.33					
SAS	February	47.8	9.81	9.13 (23)	<0.001	299	1.86	0.990
	May	34.3	8.62					
BAI	February	27.2	9.65	8.07 (23)	<0.001	300	1.65	1.000
	May	13.4	9.31					
BDI	February	23.8	8.92	6.28 (23)	<0.001	297	1.28	0.980
	May	11.8	8.67					

### 3.2 Longitudinal HRV trajectory across the training program (session‐by‐session dynamics)

**FIGURE 4:**
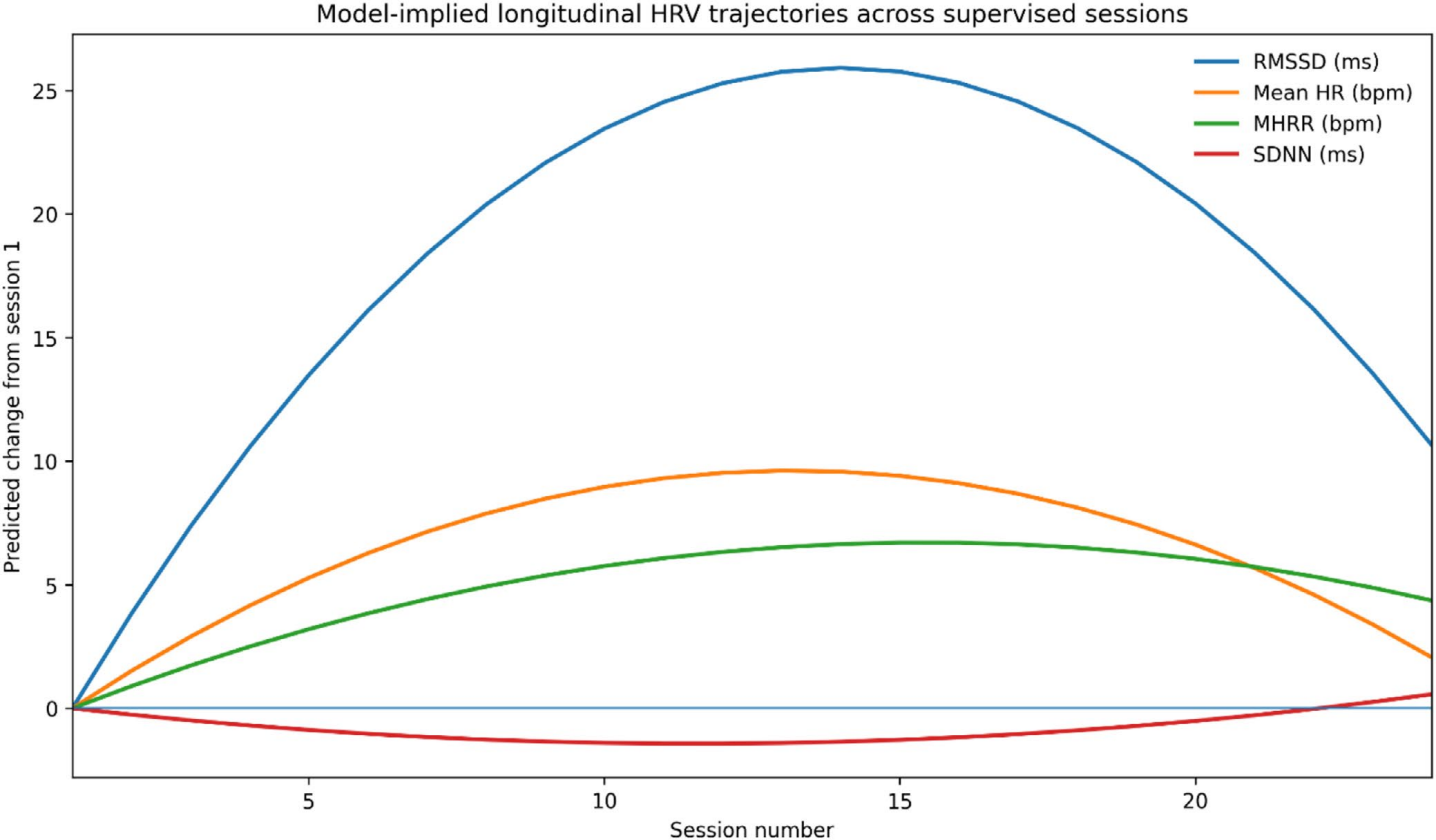
Model‐implied longitudinal trajectories of selected HRV indices across supervised HRV biofeedback sessions. Curves are reconstructed from the linear and quadratic mixed‐effects coefficients reported in Table 5 and represent the estimated population‐level trajectory across supervised sessions. This model‐based visualization was used in preference to plotting all raw repeated observations because the dataset contains a large number of within‐subject repeated measurements across the intervention period, which would result in substantial overplotting and reduced interpretability in a single figure.

Longitudinal adaptation across the supervised training period was examined separately using mixed‐effects models with linear and quadratic session terms. These analyses addressed whether HRV‐derived indices changed progressively across the repeated supervised sessions over the intervention period. Results indicated nonlinear adaptation profiles across selected autonomic measures: some indices showed no convincing cumulative trend (e.g., normalized coherence and SDNN), whereas others demonstrated statistically significant change over sessions, often with early gains followed by later stabilization or plateau‐like behavior. In particular, RMSSD and mean inter‐beat interval showed significant positive session‐related change with a quadratic component, consistent with early improvement and later stabilization; mean heart rate and mean heart rate range also showed significant nonlinear trajectories across sessions (Table 5 and Figure 4).

**TABLE 5:** Mixed‐effects model estimates of linear and quadratic session effects on HRV/coherence indices across the training period.

Outcome	Linear effect (*b*, SE, *p*)	Quadratic effect (*b* ^2^, SE, *p*)	*F*(df1, df2), *p* (linear)	*F*(df1, df2), *p* (quadratic)	Summary of trend	Approx. Peak^*^
SDNN (ms)	−0.298 (0.224), *p* = 0.184	+0.0129 (0.0089), *p* = 0.145	*F*(1,608) = 1.77, *p* = 0.184	*F*(1,1001) = 2.12, *p* = 0.145	No significant changes	‐
MHRR (bpm)	+0.992 (0.473), *p* = 0.036^*^	−0.0321 (0.0186), *p* = 0.078	*F*(1,547) = 4.40, *p* = 0.036^*^	*F*(1,1009) = 3.12, *p* = 0.078	Significant linear increase with indication of a “plateau”	Peak≈session 15–16
Mean HR (bpm)	+1.707 (0.572), *p* = 0.003^*^	−0.0647 (0.0230), *p* = 0.005^*^	*F*(1,1046) = 8.90, *p* = 0.003^*^	*F*(1,1032) = 7.88, *p* = 0.005*	Significant increase followed by slowing/decline	Peak≈session 13
RMSSD (ms)	+4.288 (1.374), *p* = 0.002^*^	−0.153 (0.053), *p* = 0.004^*^	*F*(1,464) = 9.75, *p* = 0.002^*^	*F*(1,1033) = 8.36, *p* = 0.004^*^	Significant increase reaching a plateau	Peak≈session 14

^*^ *p* < 0.05.

### 3.3 Acute within‐session physiological effects (pre → post)

Table 6 summarizes the overall acute within‐session physiological response to HRV biofeedback derived from all repeated paired pre‐session/post‐session recordings collected across the supervised training visits, rather than from any one specific supervised session. Mixed‐model analyses showed a consistent immediate physiological signature of training: normalized coherence increased significantly from pre‐ to post‐session, mean heart rate decreased, and mean inter‐beat interval increased, together indicating an acute shift toward greater parasympathetic engagement immediately after training. Mean heart rate range also decreased significantly in the immediate post‐session window. In contrast, SDNN and RMSSD did not show reliable acute pre‐to‐post changes in this pooled within‐session analysis. These acute effects should be interpreted separately from the longitudinal adaptation analyses reported below, which address whether autonomic indices changed progressively across the approximately 3‐month supervised training period. This distinction is important because the acute pre/post analysis reflects the immediate physiological response to a given training visit, whereas the longitudinal models test cumulative session‐related change over time.

**TABLE 6:** Overall acute within‐session effects derived from repeated paired pre‐session/post‐session HRV measurements across all supervised HRV biofeedback visits.

Outcome	Pre (EMM)	Post (EMM)	Post–pre difference	*p*	Interpretation of the effect
Normal coherence	81.3	83.7	+2.4	0.015^*^	Coherence significantly increased immediately after the training session
Mean heart rate (bpm)	84.6	81.0	−3.6	<0.001^*^	Heart rate significantly decreased (consistent with parasympathetic engagement)
Mean IBI (ms)	743.7	760.7	+17.0	<0.001^*^	RR interval significantly lengthened (consistent with lower HR)
Mean heart rate range (bpm)	30.0	27.6	−2.4	<0.001^*^	Heart rate range significantly narrowed, consistent with a more stable rhythm
SDNN (ms)	95.5	96.5	+1.0	0.645	No reliable effect
RMSSD (ms)	62.9	61.6	−1.3	0.634	No reliable effect

*Note*: Values are estimated marginal means (EMMs) derived from mixed‐effects models using all available paired pre‐session/post‐session recordings obtained across the supervised HRV biofeedback visits; they do not represent a single supervised session. These analyses summarize the overall acute within‐session physiological effect of training (Pre → Post), whereas longitudinal adaptation across the training period is presented separately in Table 5 and Figure 3. Thus, Table 6 reflects pooled acute within‐session effects across visits rather than longitudinal session‐by‐session change.

^*^ *p* < 0.05.

### 3.4 Cortisol and salivary IgA: Group comparisons and interpretive pattern

Comparisons of cortisol profiles indicated no statistically significant group difference in morning cortisol and in some baseline time points, but group differences emerged in post‐stressor time points during May, suggesting a more pronounced cortisol response pattern in the HRV biofeedback training group at those measurement time points (Table 7).

**TABLE 7:** Salivary cortisol concentrations by group across measurement time points: Descriptive statistics and between‐group comparisons.

Cortisol measurement time point	Control (mean ± SD, nmol/L)	Intervention (mean ± SD, nmol/L)	*t*(df)	*p*	Cohen's *d*
February (overall)	13.4 ± 7.18	15.3 ± 12.3	−0.616 (45)	0.541	−0.179
May, morning (awakening)	13.5 ± 7.86	13.6 ± 8.69	−0.077 (45)	0.939	−0.023
May, pre‐test	13.1 ± 8.89	18.2 ± 7.21	−2.174 (45)	0.035^*^	−0.634
May, 10 min post‐test	13.1 ± 7.48	20.6 ± 12.2	−2.527 (45)	0.015^*^	−0.737
May, 30 min post‐test	10.2 ± 5.83	14.6 ± 7.38	−2.265 (45)	0.028^*^	−0.661

^*^ *p* < 0.05.

In contrast, salivary IgA showed no statistically significant between‐group differences at any measurement point, although descriptive patterns suggested time‐ and context‐dependent variability that may require larger samples or longer follow‐up to resolve (Table 8).

**TABLE 8:** Salivary secretory IgA concentrations by group across measurement time points: Descriptive statistics and between‐group comparisons.

IgA measurement time point	Control (mean ± SD, μg/mL)	Intervention (mean ± SD, μg/mL)	*t*(df)	*p*	Cohen's *d*
February (overall)	163.7 ± 122.7	172.1 ± 207.8	−0.168 (45)	0.868	−0.049
May, morning (awakening)	103.2 ± 94.9	130.3 ± 150.1	−0.738 (45)	0.464	−0.215
May, pre‐test	62.2 ± 59.8	46.9 ± 41.8	1.020 (45)	0.313	0.298
May, 10 min post‐test	50.6 ± 42.3	44.0 ± 38.0	0.560 (45)	0.578	0.163
May, 30 min post‐test	43.9 ± 24.9	47.7 ± 37.9	−0.399 (45)	0.692	−0.116

### 3.5 Associations between psychological improvement and physiological change (correlational evidence)

Change scores across psychometric scales in the training group (ΔPSS, ΔSAS, ΔBAI, ΔBDI) were strongly intercorrelated, indicating coherent improvement across stress, anxiety, and depressive symptom domains (Table 9).

**TABLE 9:** Associations among changes (Δ) in psychometric scale scores (*n* = 24): Pearson and Spearman correlations.

Variable pair (Δ)	Pearson *r*	*p*	Spearman *ρ*	*p*
ΔPSS – ΔSAS	0.688	<0.001^*^	0.686	<0.001^*^
ΔPSS – ΔBAI	0.652	<0.001^*^	0.643	<0.001^*^
ΔPSS – ΔBDI	0.670	<0.001^*^	0.648	<0.001^*^
ΔSAS – ΔBAI	0.505	0.012^*^	0.476	0.019^*^
ΔSAS – ΔBDI	0.666	<0.001^*^	0.608	0.002^*^
ΔBAI – ΔBDI	0.706	<0.001^*^	0.651	<0.001^*^

^*^ *p* < 0.05.

Cortisol reactivity indices (ΔCortisol at 10 and 30 min) demonstrated statistically supported associations with improvements in anxiety‐related outcomes (particularly BAI), with a generally consistent directionality across Pearson and Spearman estimates. Additionally, mean sIgA change was negatively associated with 10‐min cortisol reactivity within the HRV biofeedback group. Because salivary IgA showed no significant between‐group differences overall, this exploratory association should not be interpreted as evidence of intervention‐related immune suppression; rather, it may reflect short‐term coupling between acute HPA‐axis reactivity and mucosal immune dynamics during the stress protocol.

Exploratory HRV–biochemistry/psychometrics associations suggested that increases in time‐domain HRV indices (e.g., RMSSD, SDNN) and software‐derived frequency‐domain LF were associated with more favorable cortisol change patterns. Additional exploratory associations emerged (e.g., HF with SAS change; coherence change with IgA change), though these findings require cautious interpretation because LF/HF estimates were secondary variables, respiration was not recorded separately, and multiple‐testing considerations apply (Table 10). Because LF and HF estimates were derived without a separately recorded respiratory signal and within the context of deliberately slowed breathing, these exploratory frequency‐domain associations should be interpreted conservatively and not as direct evidence of respiration‐independent autonomic shifts.

**TABLE 10:** Exploratory within‐group correlations among time‐domain/frequency‐domain HRV, biomarker, and psychometric change indices in the HRV biofeedback group (*n* = 24): Pearson and Spearman coefficients.

Analysis block	Variable pair (Δ)	Pearson *r*	*p*	Spearman *ρ*	*p*
Cortisol reactivity vs. symptoms	ΔCortisol_10 – ΔBAI	0.483	0.017^*^	0.509	0.011^*^
	ΔCortisol_30 – ΔBAI	0.472	0.020^*^	0.457	0.012^*^
	ΔCortisol_10 – ΔPSS	0.368	0.077	0.429	0.036^*^
Cortisol reactivity internal consistency	ΔCortisol_10 – ΔCortisol_30	0.942	<0.001^*^	0.940	<0.001^*^
IgA change vs. cortisol reactivity	ΔIgA_mean – ΔCortisol_10	−0.436	0.033^*^	−0.381	0.067
HRV‐derived indices vs. cortisol/IgA/symptoms	ΔRMSSD – ΔCortisol_before	−0.229	0.282	−0.418	0.043*
	ΔSDNN – ΔCortisol_before	−0.301	0.153	−0.426	0.039^*^
	ΔLF – ΔCortisol_before	−0.475	0.019^*^	−0.543	0.007^*^
	ΔHF – ΔSAS	0.519	0.009^*^	0.192	0.369
	ΔCoherence – ΔIgA_10	−0.723	<0.001^*^	−0.577	0.004^*^

^*^ *p* < 0.05.

These exploratory correlations describe associations among change indices within the HRV biofeedback group and should not be interpreted as direct evidence of causal intervention effects.

### 3.6 Clinically meaningful psychometric improvement by follow‐up

**FIGURE 5:**
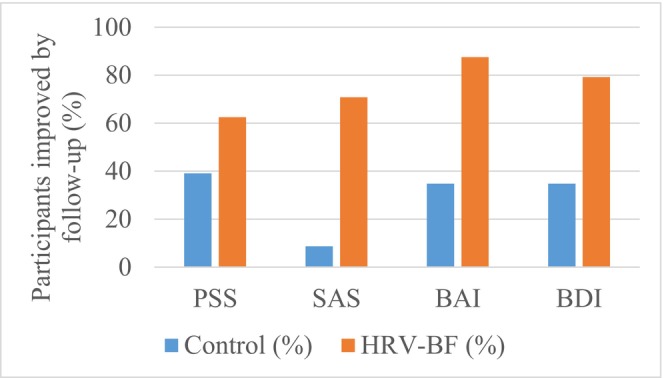
Proportion of participants achieving clinically meaningful psychometric improvement (≥20% reduction from baseline) by follow‐up (May) in the control and HRV biofeedback groups.

Because psychometric outcomes were assessed only at baseline and follow‐up, clinically meaningful improvement (defined as a ≥20% reduction from baseline) was evaluated as responder status by May rather than as continuously observed event time. Responder proportions were consistently higher in the HRV biofeedback group than in controls across all four psychometric scales: PSS, 62.5% vs. 39.1%; SAS, 70.8% vs. 8.7%; BAI, 87.5% vs. 34.8%; and BDI, 79.2% vs. 34.8% (Figure 5 and Table 11).

**TABLE 11:** Clinically meaningful psychometric improvement by follow‐up (May), defined as ≥20% reduction from baseline.

Outcome	Control n/N (%)	HRV‐BF n/N (%)
PSS	9/23 (39.1%)	15/24 (62.5%)
SAS	2/23 (8.7%)	17/24 (70.8%)
BAI	8/23 (34.8%)	21/24 (87.5%)
BDI	8/23 (34.8%)	19/24 (79.2%)

## 4 DISCUSSION

To avoid overly broad interpretation, references to autonomic regulation in the present study are limited to the specific physiological markers assessed, namely heart rate, inter‐beat interval, RMSSD, and device‐derived coherence. In addition, the term “stress” is used here either to denote perceived psychological stress assessed by psychometric instruments or the acute physiological stress response elicited by the MAST. Accordingly, salivary cortisol concentrations were interpreted as markers of HPA‐axis activation/reactivity and recovery following the acute stress challenge, rather than as inherently positive or negative outcomes in themselves.

### 4.1 Clinically meaningful symptom improvement by follow‐up

A primary finding of this research was a substantial reduction in psychological distress over the approximately 3‐month follow‐up in the HRV biofeedback group. However, because group allocation was nonrandom and the comparison group was not matched to the intervention group on baseline anxiety and depressive symptom severity, these changes should be interpreted cautiously and not as definitive evidence that HRV‐BF itself caused the observed reductions. Although pre‐post decreases were large and responder proportions were higher in the intervention group than in controls, alternative explanations cannot be excluded, including regression to the mean, natural symptom fluctuation across the semester, nonspecific expectancy/attention effects, and other uncontrolled time‐varying influences such as medication changes or mental health counseling initiated during follow‐up. In the present sample, however, no participant reported initiating anxiolytic or antidepressant treatment, and no participant reported seeking formal mental health counseling during follow‐up.

With this caution in mind, the observed pattern remains broadly consistent with previous literature indicating that HRV biofeedback is associated with reductions in self‐reported stress and anxiety, including meta‐analytic evidence reported by Goessl et al. and broader systematic reviews showing beneficial effects across emotional and stress‐related outcomes (Goessl et al., 2017; Lehrer et al., 2020). In this context, our results extend the existing evidence by showing that, in a high‐demand academic population, these improvements can be observed clearly within a structured 3‐month training period. Rather than suggesting a precise “one‐month threshold,” the present data support the interpretation that repeated HRV biofeedback practice may facilitate progressive psychophysiological self‐regulation, with symptom improvement becoming clinically evident by the end of follow‐up. This interpretation is also compatible with mechanistic accounts of HRV biofeedback, which emphasize resonance‐frequency breathing, baroreflex engagement, and strengthened autonomic self‐regulation as plausible pathways through which stress‐ and anxiety‐related symptoms may improve over time (Lalanza et al., 2023; Lehrer & Gevirtz, 2014).

### 4.2 Nonlinear autonomic adaptation: The “plateau” effect

Longitudinal modeling of the session‐by‐session HRV data revealed a nonlinear “skill acquisition” curve. Indices reflecting short‐term vagally mediated HRV, such as RMSSD and mean inter‐beat interval, followed a quadratic trajectory: they increased significantly during the first ~14 sessions before stabilizing.

This plateau effect is consistent with the law of diminishing returns in physiological training. Just as athletic conditioning reaches a maintenance phase, autonomic regulation appears to hit a “physiological ceiling” or a new homeostatic set point after about 6–8 weeks of consistent resonance frequency breathing. This mirrors findings by Wheat & Larkin (2010), who noted that the baroreflex gain—the primary mechanism targeted by HRV‐BF—improves rapidly with practice but eventually saturates (Wheat & Larkin, 2010).

Crucially, normalized coherence increased significantly within sessions (acute effect) but did not show a cumulative increase across the 3 months (chronic effect). This discrepancy highlights the difference between “state” and “trait” regulation. Students successfully entered a high‐coherence state during practice (activating the “vagal brake”), but the unrelenting academic pressure likely prevented this from translating into a permanent shift in their baseline coherence between sessions. This suggests that for this population, HRV‐BF functions more as a compensatory coping mechanism than a permanent cure for autonomic imbalance.

Because HRV biofeedback is a behavioral intervention based on paced/resonance breathing and real‐time self‐regulation rather than direct neural stimulation, acute pre‐session/post‐session changes in SDNN and RMSSD were interpreted here as session‐related physiological process measures. In the absence of a matched acute control condition, these findings should not be taken as definitive evidence of sustained vagal activation persisting beyond the training context; rather, any observed persistence is more cautiously interpreted as training‐related autonomic adaptation acquired through repeated practice.

### 4.3 Psychoneuroendocrine integration: Cortisol and immune dynamics

The study's biomarker results offer a window into the biological underpinning of the observed psychological gains. We observed a significant association between cortisol reactivity and improvements in anxiety (BAI). Specifically, the training group exhibited a more pronounced cortisol spike in response to the MAST compared to controls.

Cortisol findings should be interpreted descriptively rather than hierarchically. Although the HRV biofeedback group displayed a more pronounced salivary cortisol pattern around the MAST at follow‐up, the control group's flatter profile cannot be interpreted here as evidence of poorer stress‐system health, particularly because the control group had lower psychometric distress (Miller et al., 2007). Moreover, because the intervention group also showed higher pre‐test cortisol values, the observed differences cannot be attributed solely to the acute stress challenge itself. We therefore interpret these results as evidence of between‐group differences in cortisol dynamics under the study conditions, without inferring that one profile was inherently more adaptive than the other.

This physiological coherence was further supported by the immune data. We found a negative correlation between sIgA change and cortisol reactivity. The inverse association between ΔIgA_mean and ΔCortisol_10 was interpreted cautiously as a short‐term psychoneuroimmunological coupling pattern during the acute stress protocol, not as evidence of clinically meaningful immune suppression in the intervention group, particularly because salivary IgA showed no significant between‐group differences overall. However, the exploratory link between increased HRV (RMSSD, SDNN) and favorable cortisol patterns suggests a top‐down regulatory pathway: by strengthening vagal tone through biofeedback, students may have indirectly modulated their neuroendocrine stress sensitivity, preserving immune function during non‐stressful periods.

### 4.4 Limitations

Several limitations merit consideration. First, group allocation was nonrandom and based on baseline psychological screening, resulting in substantial baseline differences: the training group began the study with significantly higher distress scores than the control group. This design increases susceptibility to selection effects, regression to the mean, and other unmeasured confounders, thereby limiting causal inference. In addition, although the intervention and follow‐up were conducted within the active academic semester and before the start of the regular summer examination session, temporal influences related to curriculum structure, variation in academic workload across the semester, and differences between students from different academic years may still have contributed to changes in stress, anxiety, and depressive symptoms. Because participants were drawn from multiple study years, year‐specific curricular demands could not be fully standardized and remain a potential source of residual confounding. In addition, time‐varying concomitant treatments or supports during follow‐up were not systematically monitored as controlled exposures; therefore, we could not exclude influences such as initiation or modification of counseling, psychotherapy, or medication.

Second, another limitation is the marked female predominance of the sample (40/47 participants), which may limit generalizability and introduces the possibility of sex‐related confounding. Although sex distribution did not differ significantly between groups at baseline, the small number of male participants precluded meaningful sex‐stratified analyses. Furthermore, menstrual cycle phase, hormonal contraception use, and other sex hormone–related variables were not recorded; therefore, their potential influence on psychometric outcomes, autonomic measures, and salivary biomarkers could not be evaluated or adjusted for.

Third, the intervention could not be blinded and the control group did not receive an active/sham comparator. Therefore, nonspecific effects (attention, expectancy, demand characteristics) may have contributed to part of the observed improvement, particularly for self‐reported outcomes.

Fourth, no acute within‐session physiological recordings were available in the control arm because the comparator was observational and did not undergo supervised laboratory sessions; accordingly, the Pre → Post analyses should be interpreted as intervention‐arm process measures rather than as controlled between‐group acute effects.

Fifth, the study relied on supervised laboratory sessions twice weekly, and we did not electronically track the frequency or duration of home practice. Variations in home adherence could explain heterogeneity in response, with earlier responders potentially reflecting greater unsupervised practice.

Sixth, because respiration was not recorded with a separate sensor, we could not quantify respiratory rate directly or compute formal cardiorespiratory coherence; accordingly, the coherence metric reported here should be interpreted as the device‐derived HeartMath spectral coherence index rather than as respiratory–heart signal coherence.

Finally, the sample size (*n* = 47) limited precision for complex biomarker models, particularly for sIgA, which shows high inter‐individual variability and reduced power to detect small interaction effects. In addition, the follow‐up period was limited to approximately three months, so longer‐term maintenance of effects remains uncertain.

### 4.5 Practical implications for medical education

The findings of this study have direct translational value for medical curricula and student wellness programs:

1. *The “14‐Session” Standard*: Institutions implementing biofeedback should aim for a minimum of ~14 supervised sessions (approx. 7 weeks) to maximize physiological adaptation. Extending training beyond this point without changing the protocol may yield diminishing physiological returns.
2. *Program duration*: The present findings support the use of HRV biofeedback programs delivered over an approximately 3‐month period, with clear psychometric improvement evident by follow‐up and physiological adaptation emerging across repeated supervised sessions.
3. *Resilience vs. Relaxation*: Educators should frame HRV‐BF not just as “relaxation” but as “resilience training.” The goal is not to eliminate the stress response (as evidenced by the preserved cortisol reactivity) but to sharpen it—allowing students to mobilize energy when needed and recover quickly thereafter.

## 5 CONCLUSIONS

This study characterizes the physiological dynamics of HRV‐BF as a stress‐management intervention for international medical students. Our findings establish that HRV‐BF functions as a potent neurophysiological regulator that yields rapid, clinically meaningful symptom reduction. The intervention was associated with clinically meaningful psychometric improvement by follow‐up and with non‐linear autonomic adaptation across the training period, suggesting a clear dose‐related process in the acquisition of self‐regulatory skills.

Furthermore, the correlation between improved heart rate variability and restored HPA‐axis reactivity indicates that the intervention fosters biological resilience rather than mere relaxation. By strengthening the “vagal brake” and recalibrating endocrine responses to acute challenge, HRV‐BF provides students with the physiological flexibility necessary to navigate high‐pressure academic environments. These data suggest that medical institutions should implement biofeedback programs approximately 7 weeks/~14 supervised sessions to peak academic stressors to ensure students attain the necessary autonomic and psychological buffering.

## AUTHOR CONTRIBUTIONS

**Gabriela Panayotova:** Conceptualization; data curation; formal analysis; funding acquisition; investigation; methodology; project administration; resources; software; supervision; validation; visualization. **Margarita Velikova:** Funding acquisition; methodology; project administration; supervision.

## FUNDING INFORMATION

This work was supported by Fund “Science” at Medical University “Prof. Dr. Paraskev Stoyanov” – Varna, Project No. 21004, entitled “Evaluation of the therapeutic possibilities of biofeedback training as a method for reducing stress and improving the mental health of foreign medical students.”

## CONFLICT OF INTEREST STATEMENT

The authors declare that they have no potential conflict of interest.

## ETHICS STATEMENT

The study was conducted in accordance with the Declaration of Helsinki and was approved by the Committee on Ethics of Scientific Research at Medical University of Varna, Bulgaria (Protocol/Decision No. 137, 2 November 2023). All participants provided written informed consent prior to participation in the study.

## Data Availability

The data that support the findings of this study are available from the corresponding author upon reasonable request. The data are not publicly available because they contain information that could compromise the privacy of research participants and are subject to ethical restrictions.
